# Adolescent Depression and Negative Life Events, the Mediating Role of Cognitive Emotion Regulation

**DOI:** 10.1371/journal.pone.0161062

**Published:** 2016-08-29

**Authors:** Yvonne Stikkelbroek, Denise H. M. Bodden, Marloes Kleinjan, Mirjam Reijnders, Anneloes L. van Baar

**Affiliations:** 1 Child and Adolescent Studies, Utrecht University, PO Box 80.140, NL-3508 TC, Utrecht, The Netherlands; 2 Department of Developmental Psychology, Radboud University, Nijmegen, The Netherlands; 3 Trimbos Institute (Netherlands Institute of Mental Health and Addiction), Utrecht, The Netherlands; Maastricht University, NETHERLANDS

## Abstract

**Background:**

Depression during adolescence is a serious mental health problem. Difficulties in regulating evoked emotions after stressful life events are considered to lead to depression. This study examined if depressive symptoms were mediated by various cognitive emotion regulation strategies after stressful life events, more specifically, the loss of a loved one, health threats or relational challenges.

**Methods:**

We used a sample of 398 adolescents (*M*_*age*_ = 16.94, *SD* = 2.90), including 52 depressed outpatients, who all reported stressful life event(s). Path analyses in Mplus were used to test mediation, for the whole sample as well as separately for participants scoring high versus low on depression, using multigroup analyses.

**Results:**

Health threats and relational challenging stressful life events were associated with depressive symptoms, while loss was not. More frequent use of maladaptive strategies was related to more depressive symptoms. More frequent use of adaptive strategies was related to less depressive symptoms. Specific life events were associated with specific emotion regulation strategies. The relationship between challenging, stressful life events and depressive symptoms in the whole group was mediated by maladaptive strategies (self-blame, catastrophizing and rumination). No mediation effect was found for adaptive strategies.

**Conclusion:**

The association between relational challenging, stressful life events and depressive symptoms was mediated by maladaptive, cognitive emotion regulation strategies.

## Introduction

Depression during adolescence is a serious problem because of its high prevalence [1–3], considerable burden of disease [4], suicide risk [5], other comorbid psychiatric disorders [6] and the high risk of recurrence [5,7]. Although knowledge about the etiology of depression has increased in the last decade, it is still difficult to explain and predict who becomes depressed, because of the many factors involved [8]. A well-established predictor of depressive symptoms is the experience of a stressful life event [9], like a romantic break up. An intriguing question is what mechanisms are involved that may lead different types of stressful life events to contribute to the development of depressive symptoms and ultimately depression. Difficulty in emotion regulation is considered to contribute to depression [10–12]. Emotion regulation is seen as a potential mediator of depressive symptoms after stressful life events have occurred [13]. From a clinical viewpoint it is necessary to establish which factors actually mediate the occurrence of depressive symptoms after specific types of stressful life events, as such factors could then be addressed effectively in prevention and treatment efforts.

Adolescence is a challenging developmental phase with many physical and psychological changes, which may generate stress. Stressors are recognized as very important in the etiology and maintenance of internalizing problems [14]. Three different types of stressful life events have been identified, namely loss, health threats and relational challenges [15,16]. Stressful life events are associated with a larger increase in depressive symptoms than other types of stress, such as academic stress [17–22]. In children, negative life events were found to be a significant predictor of depression, putting children at risk for future depressive episodes [23,24]. Stressful life events during childhood have repeatedly been found to be associated with an increased risk of developing mental disorders during adulthood [25–27]. Furthermore, stressors can be followed by depressive symptoms, and depressive symptoms in themselves can generate stressful life events, resulting in a reciprocal relationship [14,28,29]. Garnefski and colleagues [15] found no difference in type of stressful life event and the association with depressive symptoms in adolescents aged 14 to 18. For clinically depressed adolescents, however, it is unclear as yet whether this is also true.

The dual process model of cognitive vulnerability to depression hypothesizes that associative thought processing (automatic processing) induces depressive symptoms when no correction occurs by explicitly reflective processing [30]. This is especially the case when the associative processing is negatively biased with thoughts about oneself. Furthermore, life stress appears to deplete cognitive resources, which are necessary for reflective processes to correct associative processing [30]. This dual process of self-referent association and cognitive reflection is considered to be of importance for the regulation of emotions.

Difficulty in regulating evoked emotions is often thought to lead to depression [11,12,31]. In adults, emotion regulation plays a central role in the etiology and maintenance of clinical levels of psychopathology [32–34]. Emotion regulation is a complex process with (un-)conscious, cognitive, and self-regulatory components. Emotion regulation strategies can be adaptive or maladaptive. Maladaptive emotion regulation has repeatedly been linked to various mental disorders, including the onset of depressive symptoms [35–39]. Maladaptive emotion regulation is also a risk factor for the recurrence of depression in adults [40]. Furthermore, findings suggest that the strength of the relationship between maladaptive emotion regulation strategies and psychopathology may be a function of clinical severity [41]. Some specific maladaptive cognitive emotion regulation strategies such as self-blame, rumination and catastrophizing, are associated with higher levels of depressive symptoms in adolescence, while adaptive strategies such as positive reappraisal, positive refocusing and putting things into a broader perspective, are associated with less depressive symptoms [15,42].

Only a few studies have established the relationship between stressful life events and emotion (dys)regulation in adults [10] and adolescents [13]. The type of life event influences the use of specific cognitive emotion regulation strategies, with health threat being associated with self-blame and relational challenge with other-blame in adolescence [15].

As pointed out, stressful life events have an impact on emotion regulation, which in turn influences the degree of depressive symptoms. Therefore, emotion regulation can be considered a mediator in the relationship between stressful life events and depressive symptoms [43,44]. Studies on emotion regulation as a mediator of depressive symptoms in adolescents are scarce. One study reported that emotion regulation strategies were found to mediate the relationship between interpersonal stress and depressive symptoms in undergraduate students [13]. It is unclear if this mediation is also present when adolescents have high levels of depressive symptoms, or whether the use of specific emotion regulation strategies mediates the relation between certain stressful life events and depressive symptoms.

**Fig 1:**
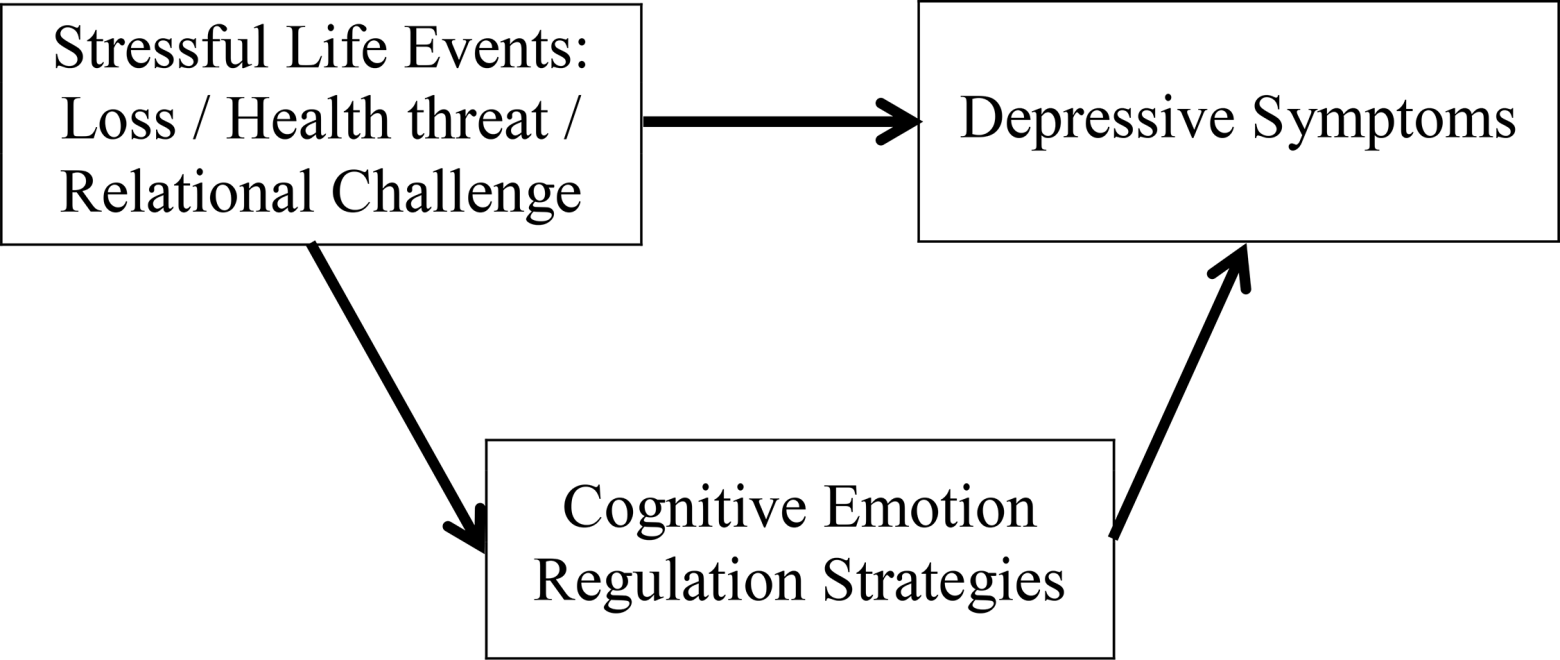
Mediation model being tested; cognitive emotion regulation strategies as mediator of depressive symptoms after a stressful life event.

The aim of this study is to examine if the relation between stressful life events and depressive symptoms is mediated by cognitive emotion regulation strategies and whether these potentially mediating effects differ per type of stressful life event (Fig 1). We used a multi-sample approach, including a community sample and clinically referred depressed outpatients. Mediation was studied for the whole sample, as well as for the depressed and non-depressed adolescents separately. We expected to find that [1] the relationship between stressful life events (loss, health threat or relational challenge) and depressive symptoms is mediated by cognitive emotion regulation strategies; [2] the model for mediation would be specific for type of stressful life event: self-blame would be important after health threat and other-blame after relational stress, and no mediation would be present after loss; [3] the model would also be specific in that the pathway from stressful life events via cognitive emotion regulation to depressive symptoms would be more pronounced in adolescents with high levels of depressive symptoms compared to adolescents with low levels of depressive symptoms.

## Methods

### Participants

In a total of 653 adolescents (of whom 84 were outpatients), those who rated no life event as stressful (*n* = 35) were excluded. Adolescents in the community sample (*N* = 569) receiving psychological treatment or social counseling (*n* = 40) were also excluded because of a potential effect on emotion regulation strategies. If answers on gender (*n* = 3), age (*n* = 4), life events or scores for whole scales were missing, cases were also excluded from the analyses (*n* = 173), which amounted to 39.2% from the community and 38.1% from the outpatient sample. Attrition analyses showed that the excluded adolescents (*n* = 255, *M*_*age*_ = 15.85, 31.8% boys) did not significantly differ from the included adolescents on the relevant scales, namely age, gender, depressive symptoms, life events and cognitive emotion regulation strategies.

The result was a final sample of 398 adolescents aged 11 to 22 years (68.8% girls, 92.5% Dutch) including 52 (13%) clinical depressed outpatients. This sample was divided into two groups with low or high scores on depression, based on the official Dutch cut-off point (<12 vs. ≥12) for clinical depression on the Children’s Depression Inventory 2 (CDI-2). These groups will be referred to as non-depressed and depressed. The non-depressed group consisted of 289 adolescents (66.8% girls, *M*_*age*_ = 16.94, 97.2% enrolled in education), whereas the depressed group consisted of 109 adolescents (74.3% girls, *M*_*age*_ = 17.09, 49.5% enrolled in education).

### Data collection

Data collection of patients was approved by the independent Medical Ethics Committee (METC) of the Utrecht Medical Centre at Utrecht University.

Two samples were used in this study. The first sample consisted of 569 adolescents (age range 11–21) from the general population who were recruited by Master's degree students in schools and sports associations across The Netherlands. The adolescents were asked to participate in the research. After written informed consent forms were obtained from the participants and their parents, a self-report questionnaire was completed.

The second sample participated in an effectiveness trial comparing Cognitive Behavioral Therapy to usual care (for more information, see [45]). 84 Clinically referred adolescents (age range 12–22) suffering from depression were recruited from 14 mental health care institutions across the Netherlands between 2011 and 2014. A psychologist informed adolescents and parents about the study and when both gave written informed consent, self-report questionnaires were completed pre-treatment using online or paper-and-pencil questionnaires.

### Measures

#### Depressive symptoms

The degree of *depressive symptoms* was measured with the Child Depression Inventory-2 [46,47], a revision of the CDI [48,49]. The CDI-2 is a self-report questionnaire for children (7 to 17 years) that reflects affective, behavioral, and cognitive symptoms of depression. Each of the 28 items offers 3 assertions: non-depressed (score 0, e.g., “I am sad once in a while”); mildly depressed (score 1, e.g., “I am sad many times.”); and clearly depressed (score 2, e.g., “I am sad all the time.”). The participants had to choose the assertion that applied most during the past two weeks. Total scores could range from 0 to 56, and a score of 12 or above (based on the CDI) is considered a clinically relevant score [49]. In this sample, 27.39% (*n* = 109) had a clinically relevant score. Reliability was very good for both the official target population of adolescents aged up to 17 years (*n* = 213, α = .93), and for adolescents older than 17 (*n* = 185, α = .91).

#### Cognitive emotion regulation strategies

*Cognitive emotion regulation strategies* were investigated with the Cognitive Emotion Regulation Questionnaire [50]. The CERQ consists of 36 items, reflecting 9 conceptually distinct adaptive or maladaptive strategies. Items refer to what someone thinks in response to a life event. The four maladaptive subscales are: Self-blame (thoughts of putting the blame of what you have experienced on yourself), Other-blame (thoughts of putting the blame of what you have experienced on others), Catastrophizing (thoughts of explicitly emphasizing the terror of an experience) and Rumination (thinking about the feelings and thoughts associated with the negative event).

The five adaptive subscales are: Putting into perspective (thoughts of playing down the seriousness of the event or emphasizing the relativity when comparing it to other events); Positive refocusing (thinking about joyful and pleasant issues instead of thinking about the actual event); Positive reappraisal (thoughts of attaching a positive meaning to the event in terms of personal growth); Acceptance (thoughts of accepting what you have experienced and resigning to yourself what has happened); and Refocus on planning (thinking about what steps to take and how to handle the negative event).

Each subscale contains 4 items measured on a 5-point Likert scale ranging from 1 (almost never) to 5 (almost always), with a higher score indicating more use of the specific adaptive strategy. Because extreme scores on Acceptance could be maladaptive the score was transformed into a categorical variable high (score above 14), low (score less than 10) and medium [36].

Research on the CERQ subscales indicated that internal consistencies were good, ranging from .67 to .81, and good validity [51]. In the present study, alphas ranged from good .70 to very good .82.

#### Stressful life events

For this study, we constructed the Life Event Scale [52], a 23 item self-report questionnaire about three types of life events, based on the distinction of psychological stress made by Lazarus [16]; Loss (1 item: death of a loved one including pets), Health threat (8 items: serious (mental) illness, suicide attempt, sexual abuse, psychological abuse, alcohol or drug abuse, crime and accidents concerning the self (not for suicide attempt), parent, sibling or friends), and Relational (or situational) challenges (14 items: parental divorce, step-parents, moving, changing schools, romantic break-up, police contact (parent or self), redundancy (parent or self), pregnancy, school failure, being bullied, conflict with parents or friends, being expelled from school). Participants were asked if they had experienced the life event, yes or no. If yes, respondents were asked to rate how stressful the event was from not stressful (0) to very stressful (3). Only adolescents with at least a score of two were included. The amount of Health threats and Relational challenges were both summed into a single score, both items were highly positively skewed. Loss was dichotomous.

### Data analytic strategy

Missing data were imputed using Relative Mean Substitution [53]. Descriptive statistics were calculated, and Pearson correlations were computed for all variables included in the models. Multivariate Analysis of Variance (MANOVA) was used to test for differences in depressive symptoms and cognitive emotion regulation strategies based on gender or on condition. To examine the correlations between the three stressful life event variables, the nine proposed mediators and the outcome measure of depression, we applied path analyses using the software package MPLUS 7 [54]. Models were tested first for the whole sample and thereafter separately for participants scoring high versus low on depression using multigroup analyses. Within the models, the correlation between the three stressful life event variables was taken into account, as well as the correlation between the proposed mediators. The comparative fit index (CFI, preferably .95 or higher), the root mean square error of approximation (RMSEA, preferably .08 or lower), and the standardized root mean square residual (SRMR, preferably .09 or lower) served as model fit indices [55].

To examine the hypothesized mediation of cognitive emotion regulation strategies in the association between the type of stressful life events and depression, we used the Model Indirect approach using MPLUS 7 with a bootstrap procedure to ensure the accuracy and robustness of the analyses and to estimate Type I errors. To assess the possible moderating effect of the level of depression, multi-group analyses were conducted within MPLUS 7. This was done by testing whether the model fit (Δχ²) was significantly better for the model in which the paths of interest were allowed to differ between non-depressed and depressed, compared to the model in which the paths of interest were constrained to be equal [56]. Next, differences between both groups for the relations between model variables were tested per direct path, also using the chi-square difference test. This was done by constraining each path of interest separately while all other paths were unconstrained, and comparing this model to the model in which the path of interest, as well as all other paths, was unconstrained. To test the differences in the indirect effects between both groups, the MODEL TEST command was used. This command permitted testing of linear restrictions on the parameters using the Wald chi-square test [54].

## Results

The percentage of adolescents with high scores (≥12) on depressive symptoms was 27.39%. The percentages of life events reported by the total sample (*N* = 398) adolescents were 24.87% for loss; 33.17% for health threats, and 60.55% for relational challenges.

A MANOVA on the total sample showed gender differences in cognitive emotion regulation strategies (*F*(9,388) = 2.754, *p* = .004), specifically girls scored lower on Other blame than boys (*F*(1,396) = 6.642, *p* = .010) and girls scored higher on Rumination than boys (*F*(1,396) = 8.548, *p* = .004). This analysis was repeated for both subgroups and also showed gender differences in the non-depressed group (*F*(9,279) = 2.441, *p* = .011), specifically girls scored lower on Other blame than boys (*F*(1,287) = 10.872, *p* = .001). An ANOVA showed no gender differences in depressive symptoms in the total group, nor in the subgroups.

A MANOVA using both the total sample and the subgroups showed no differences in cognitive emotion regulation strategies between adolescents who did or did not experience loss. An ANOVA using both the total sample and the subgroups showed no differences in depressive symptoms between adolescents who did or did not experience loss.

Another MANOVA showed group differences in the three types of stressful life events, which occurred more often in the depressed subgroup (*F*(3,394) = 24.410, *p* < .001), specifically for health threats (*F*(1,396) = 12.419, *p* < .001) and relational challenges (*F*(1,396) = 72.475, *p* < .001).

### Associations among variables

Correlations, means and standard deviations for the total sample and all model variables are reported in Table 1, and separately for depressed and non-depressed adolescents in Table 2.

**Table 1:** Correlations Total Group (*N* = 398).

			1	2	3	4	5	6	7	8	9	10	11	12	13	*M*	*SD*
**1**	**Depressive Symptoms**	***r***		**.505**	**.120**	**.430**	**.319**	**-.275**	**-.304**	**-.413**	.007	**-.278**	.049	**.254**	**.454**	9.608	8.854
		***p***		**.000**	**.016**	**.000**	**.000**	**.000**	**.000**	**.000**	.888	**.000**	.326	**.000**	**.000**		
**2**	**Self-blame**	***r***			**.222**	**.433**	**.497**	**.119**	-.086	-.004	**.296**	**.166**	.042	**.176**	**.360**	9.475	3.350
		***p***			**.000**	**.000**	**.000**	**.017**	.085	.929	**.000**	**.001**	.404	**.000**	**.000**		
**3**	**Other-blame**	***r***				**.442**	**.168**	.068	**.106**	.048	**.171**	**.124**	**-.128**	.063	**.128**	6.771	2.806
		***p***				**.000**	**.001**	.174	**.034**	.342	**.001**	**.013**	**.010**	.207	**.011**		
**4**	**Catastrophizing**	***r***					**.432**	**-.177**	-.097	**-.174**	.066	-.043	-.019	**.106**	**.276**	6.739	2.726
		***p***					**.000**	**.000**	.053	**.000**	.190	.390	.709	**.035**	**.000**		
**5**	**Rumination**	***r***						.056	-.028	**.161**	**.241**	**.323**	**.145**	**.112**	**-.141**	10.477	3.782
		***p***						.262	.574	**.001**	**.000**	**.000**	**.004**	**.026**	**.005**		
**6**	**Putting into Perspective**	***r***							**.494**	**.641**	**.452**	**.480**	.003	-.060	**-.141**	12.269	3.927
		***p***							**.000**	**.000**	**.000**	**.000**	.949	.229	**.005**		
**7**	**Positive Refocusing**	***r***								**.545**	**.330**	**.425**	-.032	-.065	**-.109**	11.912	3.870
		***p***								**.000**	**.000**	**.000**	.522	.198	**.030**		
**8**	**Positive Reappraisal**	***r***									**.400**	**.715**	-.023	-.043	-.076	12.779	3.890
		***p***									**.000**	**.000**	.648	.395	.132		
**9**	**Acceptance**	***r***										**.369**	.021	.048	**.131**	1.015	.737
		***p***										**.000**	.674	.345	**.009**		
**10**	**Refocus on Planning**	***r***											-.053	-.039	-.011	12.799	3.612
		***p***											.296	.441	.827		
**11**	**Age**	***r***												**.121**	**.143**	16.937	2.895
		***p***												**.016**	**.004**		
**12**	**Stressful Health Threats**	***r***													**.434**	.538	.919
		***p***													**.000**		
**13**	**Stressful Relational Challenges**	***r***														1.294	1.491
** **		***p***															

Significant results are printed in bold.

**Table 2:** Correlations Depressed Group (N = 109) and Non-depressed Group (N = 289).

			1	2	3	4	5	6	7	8	9	10	11	12	13	*M*	*SD*
**1**	**Depressive Symptoms**	***r***		**.372**	-.079	**.210**	**.212**	**-.218**	**-.279**	**-.340**	.061	**-.244**	-.059	**.368**	**.240**	21.743	7.721
		***p***		**.000**	.416	**.028**	**.027**	**.023**	**.003**	**.000**	.530	**.011**	.545	**.000**	**.012**		
**2**	**Self-blame**	***r***	**.181**		.095	**.384**	**.506**	.046	-.155	-.140	.158	-.017	.062	**.309**	**.271**	11.881	3.656
		***p***	**.002**		.328	**.000**	**.000**	.633	.107	.146	.100	.863	.520	**.001**	**.004**		
**3**	**Other-blame**	***r***	.078	**.228**		**.489**	**.201**	.031	.122	.110	.108	.136	-.110	-.160	.- 038	7.440	3.512
		***p***	.186	**.000**		**.000**	**.036**	.749	.207	.256	.264	.158	.254	.096	.696		
**4**	**Catastrophizing**	***r***	**.172**	**.257**	**.383**		**.464**	**-.326**	**-.202**	**-.272**	-.006	-.157	-.073	.009	.156	8.513	3.219
		***p***	**.003**	**.000**	**.000**		**.000**	**.001**	**.035**	**.004**	.950	.103	.452	.927	.106		
**5**	**Rumination**	***r***	.070	**.389**	.099	**.303**		-.050	-.124	-.013	-.038	.114	.055	.050	.155	12.330	3.687
		***p***	.237	**.000**	.092	**.000**		.607	.200	.897	.695	.238	.572	.606	.107		
**6**	**Putting into Perspective**	***r***	-.044	**.382**	**.147**	.048	**.220**		**.489**	**.650**	**.470**	**.465**	.161	.025	-.145	10.651	3.857
		***p***	.458	**.000**	**.012**	.413	**.000**		**.000**	**.000**	**.000**	**.000**	.094	.793	.133		
**7**	**Positive Refocusing**	***r***	-.063	**.141**	**.167**	**.141**	**.133**	**.445**		**.572**	**.256**	**.468**	-.055	.002	-.115	10.275	4.098
		***p***	.282	**.017**	**.005**	**.016**	**.024**	**.000**		**.000**	**.007**	**.000**	.572	.982	.234		
**8**	**Positive Reappraisal**	***r***	**-.220**	**.343**	.101	.092	**.421**	**.593**	**.470**		**.395**	**.713**	.113	-.056	-.048	10.661	3.945
		***p***	**.000**	**.000**	.086	.118	**.000**	**.000**	**.000**		**.000**	**.000**	.240	.564	.620		
**9**	**Acceptance**	***r***	-.064	**.412**	**.200**	.109	**.358**	**.470**	**.382**	**.443**		**.289**	.017	.091	.114	1.028	.726
		***p***	.276	**.000**	**.001**	.064	**.000**	**.000**	**.000**	**.000**		**.002**	.859	.348	.236		
**10**	**Refocus on Planning**	***r***	**-.178**	**.454**	**.173**	**.158**	**.528**	**.445**	**.361**	**.695**	**.413**		.136	-.092	-.078	11.551	3.463
		***p***	**.002**	**.000**	**.003**	**.007**	**.000**	**.000**	**.000**	**.000**	**.000**		.159	.340	.418		
**11**	**Age**	***r***	-.030	**.172**	-.031	**-.162**	**.270**	**.166**	-.073	**.228**	**.198**	**.195**		.092	.090	16.530	2.519
		***p***	.616	**.003**	.601	**.006**	**.000**	**.005**	.219	**.000**	**.001**	**.001**		.342	.352		
**12**	**Stressful Health Threats**	***r***	.024	-.014	.154	.062	.070	-.038	-.033	.056	.026	.041	**.161**		**.435**	.798	1.070
		***p***	.688	.815	.009	.297	.237	.520	.581	.347	.658	.493	**.006**		**.000**		
**13**	**Stressful Relational Challenges**	***r***	**.287**	**.195**	**.150**	**.129**	**.290**	.004	.057	**.130**	**.153**	**.163**	**.245**	**.384**		2.248	1.701
		***p***	**.000**	**.001**	**.011**	**.028**	**.000**	.942	.337	**.027**	**.009**	**.005**	**.000**	**.000**			
	***M***		5.031	8.568	6.519	6.069	9.779	12.879	12.529	13.578	1.010	13.270	17.090	.439	.934		
** **	***SD***	*** ***	2.990	2.728	2.626	2.172	3.581	3.784	3.599	3.561	.743	3.560	3.015	.836	1.227		

The depressed sample is displayed in the top half of the table and the non-depressed sample is displayed in the bottom half; significant results are printed in bold.

Within the *total sample*, age did not correlate significantly with depressive symptoms. Health threatening and relational challenging stressful life events showed weak to moderate correlations with depressive symptoms. Except for acceptance, all cognitive emotion regulation strategies, correlated weakly to strongly with the number of depressive symptoms. Weak to moderate correlations were found for relational challenging stressful life events and seven out of nine cognitive emotion regulation strategies; self-blame, other-blame, catastrophizing, rumination, putting into perspective, positive refocusing and acceptance.

Within the *non-depressed group*, age did not correlate significantly with depressive symptoms. Relational challenging stressful life events were weakly correlated with more depressive symptoms. Weak correlations between relational challenging stressful life events and several strategies were found, namely; self-blame, other-blame, catastrophizing, rumination, positive reappraisal, acceptance and refocus on planning. Of these strategies, only self-blame, catastrophizing, positive reappraisal and refocus on planning in turn correlated weakly with depressive symptoms. Stressful health threatening life events correlated weakly with other-blame, which did not significantly correlate with depressive symptoms.

In the *depressed group*, age did not correlate significantly with depressive symptoms. Relational challenging and also health threatening stressful life events showed a weak to moderate correlation with the degree of depressive symptoms, as well as with self-blame. All strategies, except for other-blame and acceptance, were weak to moderately correlated with depression.

### Path analyses

**Fig 2:**
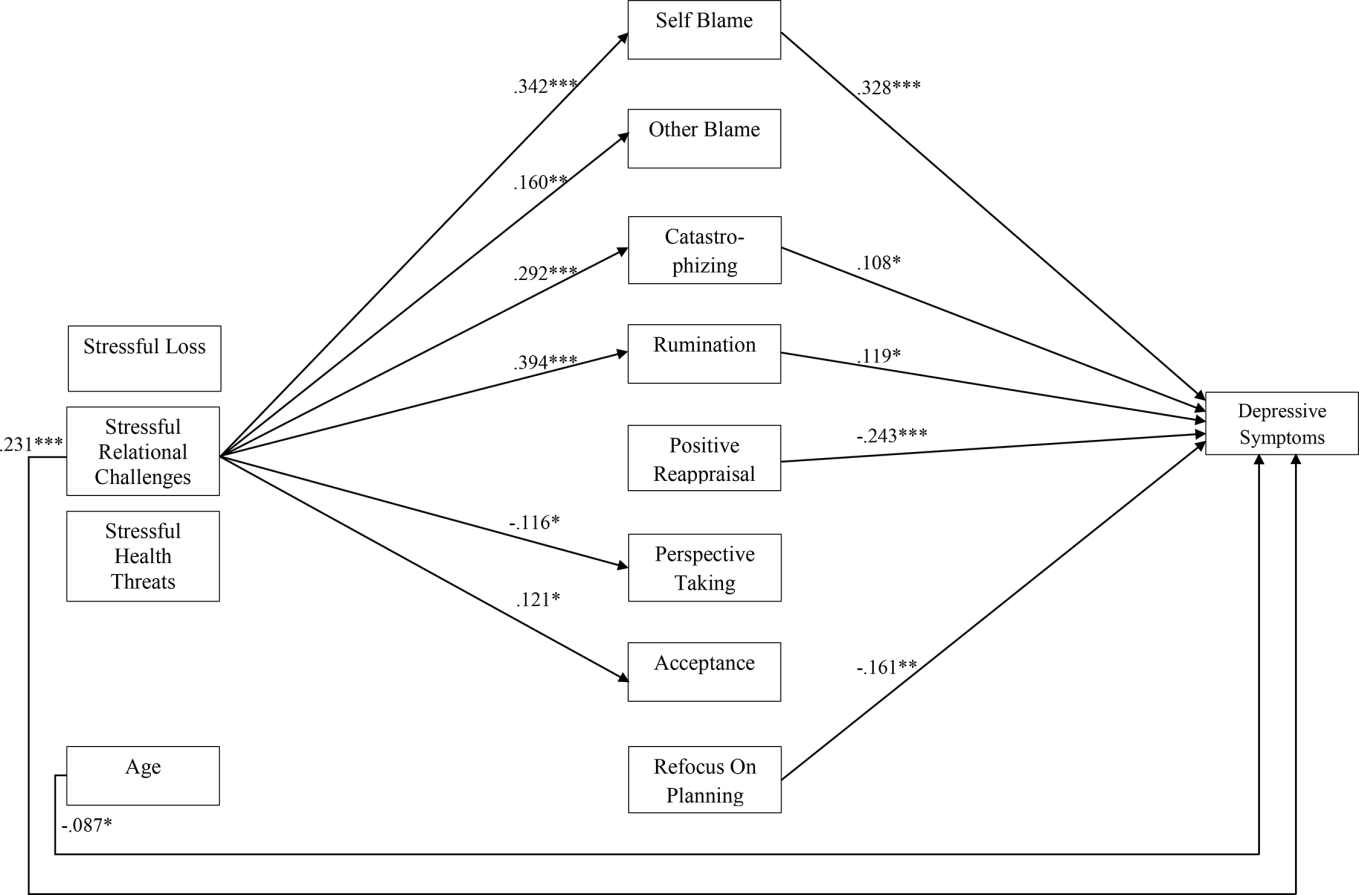
Direct Effects for the Total Sample. Standardized estimates of the direct effects on the cognitive emotion regulation and depressive symptoms. Only significant effects (* *p* < .05, ** *p* < .01, *** *p* < .001) within the total sample (*N* = 398) are shown.

**Fig 3:**
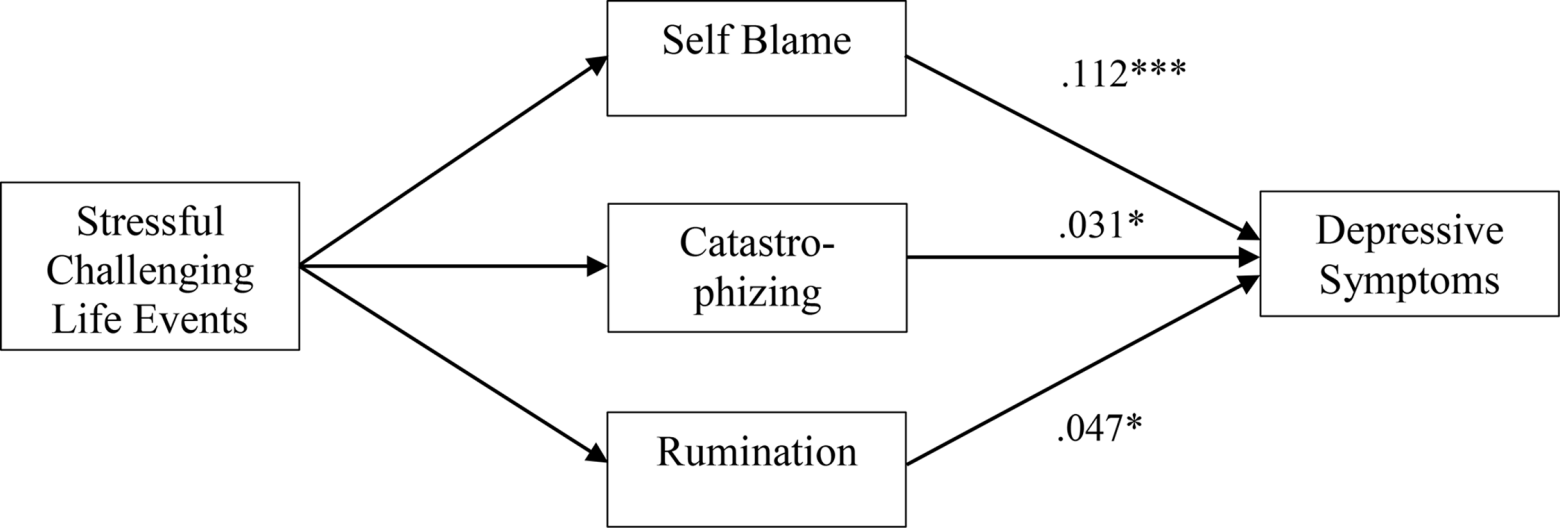
Mediation Model. Standardized estimates of the indirect effects on the depressive symptoms (N = 398). Only significant effects are shown (* *p* < .05, *** *p* < .001). Estimates apply to the total sample (*N* = 398).

The model fit indices for the mediation model within the whole sample were satisfactory (CFI = .95, RMSEA = .09, SRMR = .05). Multi-group analysis was used to test differences in depression level. The Chi-squared difference test indicated that the model differed for non-depressed and depressed participants (Δχ² (80) = 158.45, *p* < .001). The results of the model are described for participants with high and low depression scores separately below (see Fig 2 for the model and Fig 3 for the indirect effects). Gender was included in the model, but showed no direct significant effects on depressive symptoms or on any cognitive emotion regulation strategy.

#### Direct associations between type of life events and depression

Within the total sample and within the non-depressed subgroup, only relational challenging stressful life events were associated with depressive symptoms (β = .231, *p* < .001 and β = 338, *p* < .001, respectively). The depressed group showed a significant association of health threatening stressful life events with depressive symptoms (β = .285, *p* = .003). This association was also significantly different compared to the non-depressed group (Δχ² (1) = 11.55, *p* < .001). Loss was not associated with depressive symptoms in the total sample, or in either subgroup.

#### Direct associations between type of life events and cognitive emotion regulation strategies

Within the total sample, relational challenging stressful life events were associated with various strategies, namely self-blame, other-blame, catastrophizing, rumination, putting into perspective and acceptance (Fig 2). Within the non-depressed group, direct associations between relational challenging stressful life events and other-blame (β = .122, *p* = .039), positive reappraisal (β = .130, *p* = .034) and refocus on planning (β = .168, *p* = .004). Within the depressed group, direct associations between relational challenging stressful life events and self-blame (β = .195, *p* = .049), catastrophizing (β = .209, *p* = .041) and rumination (β = .240, *p* = .011) were found.

Health threatening stressful life events were not significantly associated with any cognitive emotion regulation strategy within the whole sample, nor within the subgroups. Loss was negatively associated with catastrophizing within the non-depressed group (β = -.136, *p* = .015).

#### Direct associations between cognitive emotion regulation strategies and depression

Within the whole sample more depressive symptoms were significantly associated with: self-blame, catastrophizing, rumination, positive reappraisal and refocus on planning (Fig 2). In the non-depressed group, self-blame (β = .252, *p* < .001), less positive reappraisal (β = -.269, *p* = .003) and less refocusing on planning (β = -.245, *p* = .004) were significantly associated with depressive symptoms. In the depressed group, no significant associations between degree of depressive symptoms and use of any cognitive emotion regulation strategy was found. The correlations between strategies and depressive symptoms did not differ significantly between depressed and non-depressed subgroup. Acceptance was not significantly associated with the degree of depressive symptoms within either subgroup.

#### Indirect associations

Within the whole sample, significant indirect paths from relational challenging stressful life events to depressive symptoms were found via self-blame, catastrophizing and rumination (Fig 3).

Indirect paths were found in the non-depressed group, from relational challenging stressful life events to depressive symptoms via self-blame and refocusing on planning. Within the depressed group, no indirect paths were identified. However, the Wald test of parameter constraints showed no significant differences between the non-depressed and depressed group for these indirect paths.

## Discussion

The findings of this study support the general hypothesis that certain stressful life events were related to the level of depressive symptoms and that this relationship was mediated by maladaptive cognitive emotion regulation strategies. These findings are important for clinical practice to increase understanding of the association between specific types of stressful life events and the use of cognitive emotion regulation strategies. In addition, the results confirm the dual process model on associative and reflective processing in adolescents and depression [30]. The important role of self-referent association, maladaptive cognitive emotion regulation strategy namely self-blame, as a mediator of depressive symptoms was confirmed.

The main results can be summarized as follows: (I) Health threatening and relational challenging stressful life events were associated with depressive symptoms, while stressful loss related life events were not; (II) more frequent use of maladaptive cognitive emotion regulation strategies was related to more depressive symptoms; (III) more frequent use of adaptive cognitive emotion regulation strategies was related to less depressive symptoms; (IV) specific life events were associated with specific emotion regulation strategies; (V) only the association between relational challenging stressful life events and depressive symptoms was mediated by maladaptive cognitive emotion regulation strategies (self-blame, catastrophizing and rumination); and (VI) adaptive cognitive emotion regulation strategies were not identified as mediators in the total sample.

These results deserve some further interpretation and reflection. The relationship between cognitive emotion regulation strategies and depressive symptoms was established in our study. More use of maladaptive (self-blame, catastrophizing, rumination) and less use of adaptive cognitive emotion regulation adaptive strategies (positive reappraisal, refocus on planning) were significantly associated with more depressive symptoms in the whole sample. This finding is in line with earlier findings that maladaptive emotion regulation was linked to the onset of depressive symptoms [35–39]. Maladaptive cognitive emotion regulation could representative of the self-referent association of the dual process model and adaptive cognitive reflection is considered to be of importance for the regulation of emotions.

Deficient emotion regulation is also a risk factor for recurrence of depression [40]. Our findings suggest that using maladaptive strategies can be more harmful, than the absence of using adaptive strategies. These findings are relevant for clinical practice to enhance prevention and treatment in order to detect and address specific mechanisms at work in depression.

One specific and adaptive cognitive emotion regulation strategy, acceptance, was not significantly associated with depressive symptoms, as was also reported in a meta-analysis band colleagues [41]. Increase of acceptance of a problem or risk is a common objective in various treatments such as Mindfulness, and Acceptance and Commitment Therapy [57]. In acceptance-based treatments, acceptance is promoted in order to reduce experiential avoidance [58,59]. However, the role of acceptance might differ during the process of handling stressful life events and therefore it might also have been disguised in our study. A high score on acceptance immediately after a stressful life event could, for instance, be related to learned helplessness. Timing in relation to the occurrence of stressful life events should be taken into account in future research on acceptance. In addition, acceptance may be more of an end state that is based upon other regulation strategies, instead of reflecting an active and dynamic cognitive emotion regulation strategy.

Stressful loss was not found to be associated with higher levels of depressive symptoms. This finding that the death of a loved one was generally not associated with elevated levels of depressive symptoms should be interpreted with caution. Earlier studies found that a substantial number of 75% to 80% of children do not develop mental health problems after the death of a parent or sibling, but these studies also found a significant increase in internalizing symptoms in these cases [60–64]. Another study found a significant increase in depression in the second year after bereavement [65]. In our study however, lapse of time after loss could not be accounted for. In a community sample, the comparison between family bereaved and non-bereaved showed a robust difference in internalizing problems by the age of 19 [24]. No long-term effects could be assessed in the current study. Furthermore, a broad definition of loss was used, which included pets. The kind of loss might also be of importance for the impact on mental health.

Health threatening stressful life events were only associated with depressive symptoms in the depressed group and not in the non-depressed group. Although the level of depressive symptoms was high in the depressed adolescents, health threats still accounted for substantially more depressive symptoms. These findings are in line with a review on depressive symptoms in epileptic youth, showing an elevated risk for depression in this specific group with health problems [66]. So it seems that health threats are particularly important for depressive symptoms in adolescents.

An association between relational challenging stressful life events and depressive symptoms was established for the total sample, and seen in both the depressed as well as the non-depressed group. This association was not found in earlier research conducted with secondary school students [67]. This discrepancy can be explained by the use of a larger multi-group sample in this study with higher levels of depressive symptoms, which made detection of the association possible. This shows that the use of multiple samples is indeed important in future research on cognitive emotion regulation [41].

Our findings confirm the existence of a specific association between relational challenges and cognitive emotion regulation. Loss or health threatening stressful life events showed no specific association with any of the cognitive emotion regulation strategies within the whole group, suggesting that the type of stressful event influenced the use of specific cognitive emotion regulation. Relational challenging stressful life events were associated with maladaptive strategies as well as with two adaptive strategies: putting experiences into perspective and acceptance. The non-depressed group showed a significant association between relational challenging stressful life events and three adaptive strategies, namely positive reappraisal, refocus on planning, and acceptance. In the depressed group, this association was not found, suggesting that non-depressed and depressed adolescents differ in their use of maladaptive and adaptive cognitive emotion regulation strategies. However, no significant difference between groups in the strength of this association was found. These results must thus be interpreted with caution, and studies with larger groups are needed to rule out power issues in interpreting these differences.

The mediating role of maladaptive cognitive emotion regulation strategies was established, Self-blame, catastrophizing and rumination could be identified as mediators between stressful relational challenging life events and depressive symptoms. However, depressed and non-depressed adolescents did not differ significantly in these mediation relationships, which may be due to the size of the depressed sample. Still, this is an important finding, which could be useful for clinical practice. Experiencing relational challenging stressful life events and blaming oneself, emphasizing the terror of experiences or dwelling on feelings and thoughts about the events, may put adolescents at risk for depressive symptoms.

### Strengths and limitations

This study is innovative for several reasons. First, according to the literature, testing mediational models on the etiology of adolescent depression was needed [14]. Second, the use of the multi-sample approach, including severely depressed adolescent patients, is scarce and should be used more often [68], as it is essential for the study of psychopathology [14,41]. Third, a rigorous criterion for life events was used, namely stressful life events reported by the adolescent as upsetting. Previous research used the number of life events as a variable, without the upsetting criterion, thereby ignoring whether the life events actually impact the life of participants in a negative fashion. Fourth, not only being upset but also the type of life event was taken into account as an important variable affecting depressive symptoms, as well as the cognitive emotion regulation strategies. Fifth, to our knowledge, we were the first to test the mediating role of cognitive emotion regulation in the association between stressful life events and depressive symptoms in adolescents and to test differences in mediation between low and high levels of depressive symptoms.

This study also has several limitations. First, cross-sectional data were used and therefore no temporal conclusions could be drawn. Future research is needed to test mediational models in a longitudinal design. Second, the profile of different mediators within one individual could not be taken into account. For example, the mediation of depressive symptoms by rumination may be more prominent if the use of self-blame is high and positive refocusing is low. Even more useful for clinical practice would be the identification of patterns in how the various emotion regulation strategies are used [69].

Third, the relationship between associative (maladaptive strategies) and reflective processing (adaptive strategies) could not be taken into account, while this may be a function of clinical severity [41]. Further research on specific correlations between cognitive emotion regulation strategies is needed and of importance to determine which strategy should be addressed in interventions.

Fourth, a lack of power could underlie the fact that no mediating paths were found in the depressed group, and that no significant differences in the strength of the mediational paths between the non-depressed and depressed adolescents were found. A larger sample than N = 109 is needed to identify possible mediators in the depressed group.

Fifth, the time between the last stressful life event and the measurement of depressive symptoms was not taken into account. In a longitudinal design this variable should be included because depressive symptoms could increase instantly or gradually after some time, through use of specific cognitive regulation strategies [70].

Despite the mentioned limitations, our study contributes to current scientific knowledge by showing that depressive symptoms are mediated by maladaptive cognitive emotion regulation strategies (self-blame, rumination and catastrophizing) uniquely after stressful relational challenging life events. Mediation was not found after losing a loved one or experiencing a health threat. Adaptive cognitive emotion regulation strategies, for instance acceptance, were not identified as mediators. These findings are important for clinical practice. Use of specific maladaptive cognitive regulation strategies after relationally challenging stressful life events can aggravate depressive symptoms. To prevent depression after negative life events, maladaptive cognitive emotion regulation strategies should be reduced in adolescents.
