# Correction: Adolescent Depression and Negative Life Events, the Mediating Role of Cognitive Emotion Regulation

**DOI:** 10.1371/journal.pone.0192300

**Published:** 2018-01-30

**Authors:** Yvonne Stikkelbroek, Denise H. M. Bodden, Marloes Kleinjan, Mirjam Reijnders, Anneloes L. van Baar

In the Data Availability section, an incorrect DANS database URL is listed. The correct URL is: http://dx.doi.org/10.17026/dans-zg6-9jrk.
